# The novel E-subgroup pentatricopeptide repeat protein DEK55 is responsible for RNA editing at multiple sites and for the splicing of *nad1* and *nad4* in maize

**DOI:** 10.1186/s12870-020-02765-x

**Published:** 2020-12-09

**Authors:** Ru Chang Ren, Xu Wei Yan, Ya Jie Zhao, Yi Ming Wei, Xiaoduo Lu, Jie Zang, Jia Wen Wu, Guang Ming Zheng, Xin Hua Ding, Xian Sheng Zhang, Xiang Yu Zhao

**Affiliations:** 1 State Key Laboratory of Crop Biology, College of Life Sciences, Shandong Agricultural University, Taian, Shandong 271018 PR China; 2 Institute of Molecular Breeding for Maize, Qilu Normal University, Jinan, 250200 PR China; 3 State Key Laboratory of Crop Biology, College of Plant Protection, Shandong Agricultural University, Taian, Shandong 271018 PR China

**Keywords:** Defective kernel, Maize, Mitochondrion, Pentatricopeptide repeat proteins, RNA processing, Splicing

## Abstract

**Background:**

Pentatricopeptide repeat (PPR) proteins compose a large protein family whose members are involved in both RNA processing in organelles and plant growth. Previous reports have shown that E-subgroup PPR proteins are involved in RNA editing. However, the additional functions and roles of the E-subgroup PPR proteins are unknown.

**Results:**

In this study, we developed and identified a new maize kernel mutant with arrested embryo and endosperm development, i.e., *defective kernel* (*dek*) *55* (*dek55*). Genetic and molecular evidence suggested that the defective kernels resulted from a mononucleotide alteration (C to T) at + 449 bp within the open reading frame (ORF) of Zm00001d014471 (hereafter referred to as *DEK55*). *DEK55* encodes an E-subgroup PPR protein within the mitochondria. Molecular analyses showed that the editing percentage of 24 RNA editing sites decreased and that of seven RNA editing sites increased in *dek55* kernels, the sites of which were distributed across 14 mitochondrial gene transcripts. Moreover, the splicing efficiency of *nad1* introns 1 and 4 and *nad4* intron 1 significantly decreased in *dek55* compared with the wild type (WT). These results indicate that DEK55 plays a crucial role in RNA editing at multiple sites as well as in the splicing of *nad1* and *nad4* introns. Mutation in the *DEK55* gene led to the dysfunction of mitochondrial complex I. Moreover, yeast two-hybrid assays showed that DEK55 interacts with two multiple organellar RNA-editing factors (MORFs), i.e., ZmMORF1 (Zm00001d049043) and ZmMORF8 (Zm00001d048291).

**Conclusions:**

Our results demonstrated that a mutation in the *DEK55* gene affects the mitochondrial function essential for maize kernel development. Our results also provide novel insight into the molecular functions of E-subgroup PPR proteins involved in plant organellar RNA processing.

**Supplementary Information:**

The online version contains supplementary material available at 10.1186/s12870-020-02765-x.

## Background

Pentatricopeptide repeat (PPR) proteins compose a large protein family found in most land plants, with more than 450 members identified in *Arabidopsis thaliana*, *Oryza sativa*, and *Zea mays* [1–5]. These proteins contain standard tandem degenerate repeat motifs, which form a helix-loop-helix structure of approximately 35 amino acids. PPR proteins are classified mainly into P- and PLS-type subfamilies according to their PPR repeat motifs [2, 6, 7]. P-subfamily PPR proteins contain only classic “P” motif repeats in tandem, while PLS-subfamily PPR proteins contain alternating repeats of three PPR motifs of different lengths. The latter are usually divided into PLS, E, E+, and DYW subgroups based on the presence of E, E+, or DYW domains at the carboxy-terminal end [2]. A new class of PPR proteins that contain small MutS-related domains at the carboxy-terminal end has also been identified, [8, 9].

P-type PPRs are considered to be involved in group II intron splicing, RNA stabilization, cleavage, translational activation, and transcript accumulation. In contrast, PLS-type PPRs play essential roles in the conversion of cytidine (C) to uridine (U) at specific sites of organelle transcripts [4, 10, 11]. Most plant PPR proteins are targeted to mitochondria, chloroplasts, or both and regulate the functions and development of those organelles [10]. In the mitochondria, the oxidative phosphorylation system comprises five complexes (I-V) [12]. Normal assembly of these complexes is essential to maintain mitochondrial function, which requires the standard processing of mitochondrial pre-mRNAs, involving both RNA editing and intron splicing [13, 14]. Numerous PPRs are responsible for RNA posttranscriptional processes in mitochondria [15–21].

E-subgroup PPR proteins (e.g., slow growth 1 (SLO1), organelle transcript processing 87 (OTP87), mitochondrial editing factor 3 (MEF3), MEF9, MEF12, and mitochondrial PPR 25 (MPR25)) play vital roles in mitochondrial RNA editing and plant development [22–27]. In addition, several E-subgroup proteins in *Arabidopsis* and rice are involved in RNA splicing [28, 29]. In maize, five E-subgroup PPR proteins have been characterized, and all of them are involved in RNA editing. SMALL KERNEL (SMK) 1 (SMK1) is critical for *nad7*–836 editing in mitochondria and is conserved in maize and rice [14]. SMK4 is critical for RNA editing of *cytochrome c oxidase 1* (*cox1*) at position + 1489 bp [30], *ccmF*_*N*_ is essential for cytochrome c maturation, and EMPTY PERICARP 7 (EMP7) is responsible for its editing at the + 1553 bp position [31]. DEK39 is necessary for RNA editing of *nad3* transcripts in mitochondria [32], and Dek10 is responsible for RNA editing of three sites of *nad3* and *cox2* transcripts [33]. However, it is still unclear whether E-subgroup PPRs are involved in RNA editing and intron splicing in maize organelles.

Here, we identified maize mutant *dek55*, which has an embryo-lethal phenotype and arrested endosperm development, which is caused by a mutation of the mitochondrion-localized E-subgroup PPR protein DEK55. In the *dek55* mutant, the splicing efficiency of *nad1* introns 1 and 4 *trans-*splicing and *nad4* intron 1 *cis*-splicing decreased. Moreover, the editing percentages of 24 editing sites (*atp1*–1490, *atp8*–123, *ccmFc*-160, *ccmFc*-799, *ccmFc*-866, *ccmFc*-906, *ccmFc*-1144, *ccmFc*-1244, *ccmFn*-287, *ccmFn*-302, *cob*-564, *mat-r*-1877, *nad3*–146, *nad3*–190, *nad4*–77, *nad6*–25, *nad6*–138, *nad6*–146, *nad6*–159, *nad6*–161, *rps12-ct*-418, *rps12*–284, *ribosomal protein S13* (*rps13*)-56, and *rps3*–69) was also substantially reduced. Further, DEK55 could interact with ZmMORF1 and ZmMORF8 in yeast, which might be responsible for the activity of DEK55 on multiple editing sites. Taken together, our results suggest that the E-subgroup PPR protein DEK55 is involved in both RNA editing and group II intron splicing in maize mitochondria.

## Results

### Genetic and phenotypic analysis of the *defective kernel 55–1* (*dek55–1*) mutant

**Fig. 1:**
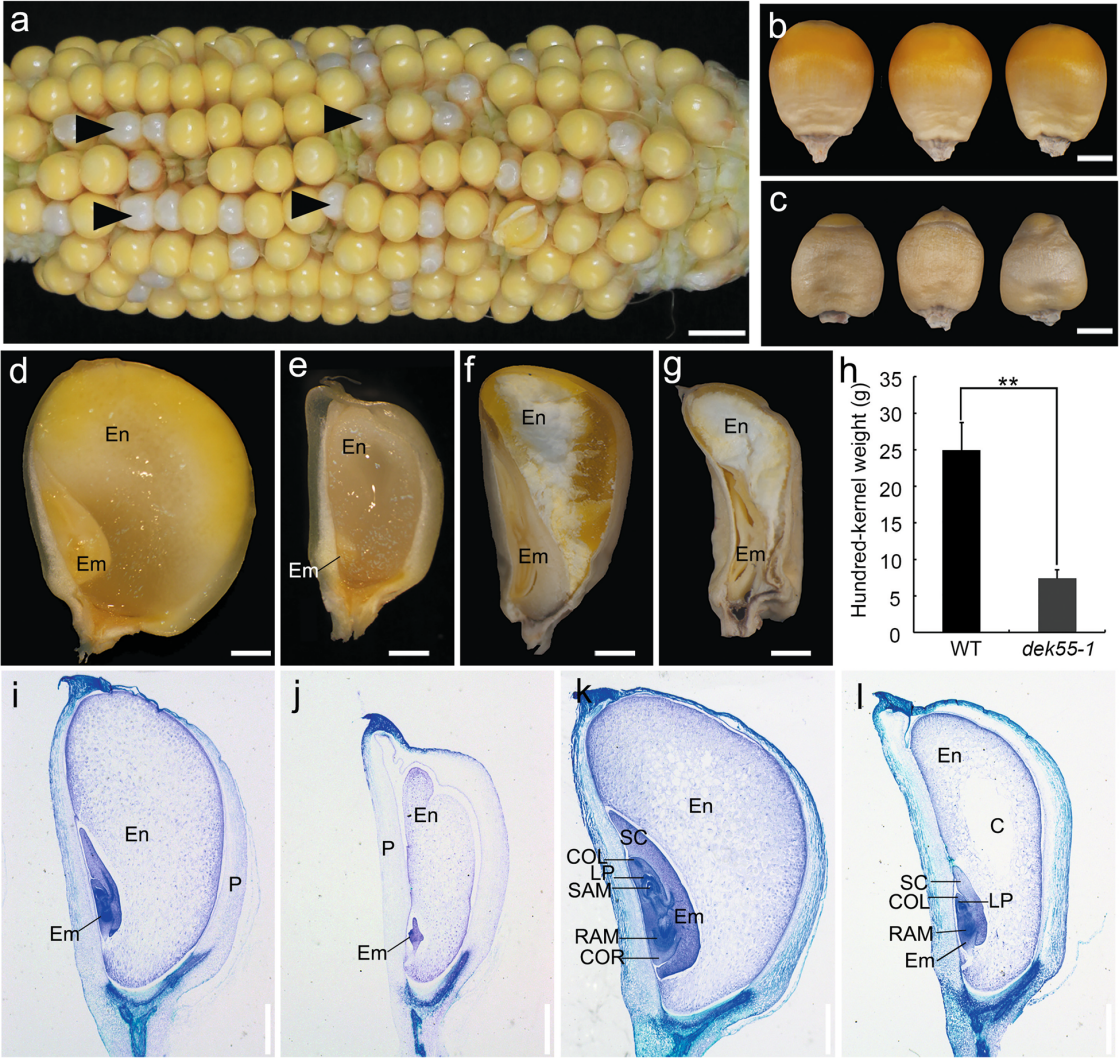
Phenotypic characterization of *dek55–1* kernels. **a** Self-pollinated *dek55–1* heterozygous ears at 15 DAP. Several mutant kernels are indicated with arrowheads. Scale bars = 1 cm. **b-c** Mature kernels of WT and *dek55–1* plants. **b**. WT. **c**, *dek55–1*. Scale bars = 2 mm. **d-g** Comparative anatomy of WT and *dek55–1* kernels at 15 DAP and at maturity. **d** and **f** WT kernels. **e** and **g** *dek55–1* kernels. Scale bars = 1 mm. **h** Hundred-kernel weight of WT and *dek55–1* kernels at maturity. (The asterisks indicate significant differences; **, *P* < 0.05, Student’s *t*-test.). **i-l** Histological analysis of WT and *dek55–1* kernels at 12 and 18 DAP. **i** and **k** WT at 12 and 18 DAP, respectively. **j** and **l** *dek55–1* kernels at 12 and 18 DAP, respectively. Scale bars = 1 mm. En, endosperm; Em, embryo; P, pericarp; LP, leaf primordium; RAM, root apical meristem; SAM, shoot apical meristem; SC, scutellum; COL, coleoptile; COR, coleorhiza; C, cavity

A mutant with a defective kernel phenotype was isolated from an ethylmethanesulfonate-induced maize B73 background population and was subsequently named *dek55–1*. The *dek55–1* kernels segregated from self-pollinated progeny of *dek55–1/+* heterozygotes at a 1:3 mendelian ratio (Fig. 1a, Additional file 1: Table S1). These results suggested that *dek55–1*, which exhibits a recessive phenotype, is caused by a monogenic mutation, which was confirmed in other populations generated from *dek55–1/+* heterozygotes crossed with C733 or S162 inbred lines (Additional file 1: Table S1).

The *dek55–1* kernels were smaller and presented a whitish pericarp, and they could be distinguished from the wild-type (WT) kernels at 15 days after pollination (DAP) (Fig. 1a). At maturity, the *dek55–1* kernels were much smaller and shrivelled (Fig. 1b, c). To further determine the mutant phenotype, both WT and *dek55–1* kernels were longitudinally sliced at 15 DAP. Compared to the WT kernels, the mutant kernels had a small, soft endosperm. (Fig. 1d, e). Furthermore, compared with the WT kernels, the *dek55–1* kernels contained a smaller mature embryo and a reduced proportion of hard endosperm (Fig. 1f, g). In addition, the weight of *dek55–1* kernel was approximately 70% lower than that of WT kernels (Fig. 1h). No *dek55–1* seeds (0/100) germinated under field conditions, implying that embryo arrest is lethal in the mutants.

To further investigate the developmental structure of *dek55–1* kernels, we examined the kernel tissue structure of the WT and *dek55–1* mutant at 12 and 18 DAP (Fig. 1i-l). At 12 DAP, the *dek55–1* embryo had only a small scutellum whose development was arrested at the coleoptile stage and a large interspace between the endosperm and the seed coat. In contrast, the WT embryo contained a visible coleoptile, a shoot apical meristem, a scutellum, and two leaf primordia, and the kernel was filled with endosperm cells (Fig. 1i, j). At 18 DAP, the WT embryo had developed complete structures, including four leaf primordia, a shoot apical meristem, and a clearly visible root apical meristem (Fig. 1k), while the *dek55–1* embryos presented only a single leaf primordium (Fig. 1l). In addition, fewer starch grains accumulated in the *dek55–1* endosperm cells than in the WT endosperm cells at this stage (Fig. 1k, l), and a cavity was observed in the *dek55–1* endosperm (Fig. 1l). Taken together, these results indicate that developmental defects in the embryo and endosperm had occurred in the *dek55–1* mutant.

### Map-based cloning of *DEK55*

**Fig. 2:**
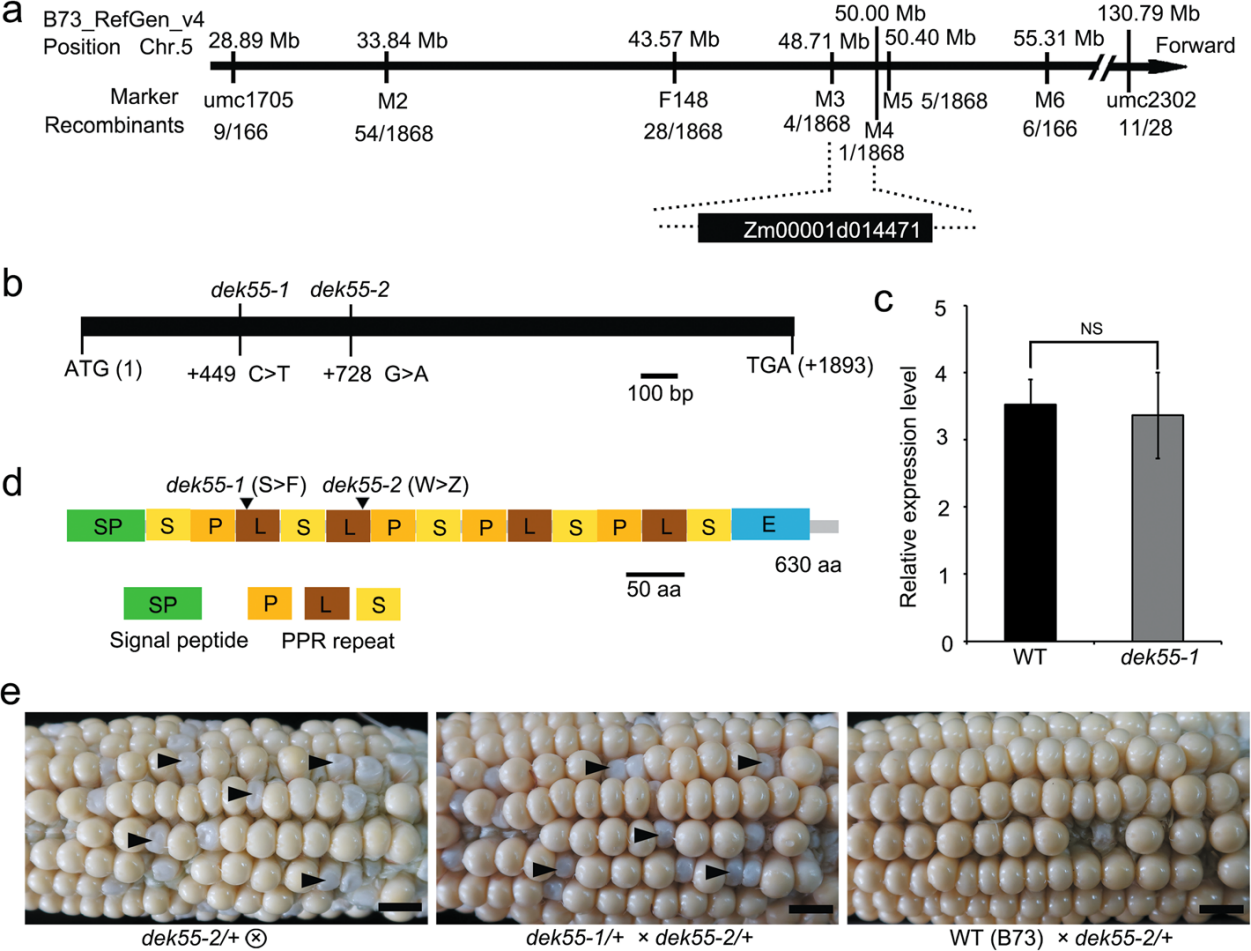
Map-based cloning and identification of *DEK55.* **a** Fine mapping of the *DEK55* locus. The *DEK55* locus was mapped to a 1.29 Mb region between marker 3 (M3) and M4 on chromosome 5, in which there are 25 candidate genes. The physical location of polymorphic molecular markers and the number of recombinants are shown in the schematic diagram. **b** Schematic structure of the *dek55* gene. The mutation sites of *dek55–1* and *dek55–2* are shown. **c** Relative expression level of *DEK55* in WT and *dek55–1* kernels. The values are the means of three biological replicates. The error bars represent the standard deviations. (Not significant (NS); *P* > 0.05, Student’s *t*-test). **d** Schematic diagram of the DEK55 protein, which contains a total of 13 PPR domains (P, L and S) and an E domain. The amino acid changes in *dek55–1* and *dek55–2* are indicated. **e** The self-pollinated *dek55–2*/+ (heterozygote) at 15 DAP, *dek55–1/+* and *dek55–2/+* were used in an allelism test of *dek55*. A cross between *dek55–2*/+ with B73 (WT) was used as a control. Several mutant kernels are indicated by the black arrowheads. Scale bars = 1 cm

To identify the *DEK55* gene, we applied the classic map-based cloning strategy to identify filial 2 (F_2_) mutant kernels, which segregated from a self-pollinated F_1_ hybrid ear. Four genomic DNA pools (10 mutant kernels per pool) and the DNA of both of the parents were used to identify the chromosomal location of the *DEK55* gene. Six simple sequence repeat (SSR) markers on chromosome 5 were strongly correlated with defective kernel phenotypes, suggesting that the candidate gene may be on chromosome 5. Further analysis showed that the *DEK55* gene is located between umc1705 and umc2302 on chromosome 5 (Fig. 2a). One thousand eight hundred and sixty-eight mutant kernels from the F_2_ population were genotyped to narrow the gene location by the use of six polymorphic molecular markers. The *DEK55* gene was ultimately located on an approximately 1.29 Mb region between molecular marker 3 (M3) and M4 (Fig. 2a). There are 25 putative protein-coding genes in this region (http://ensembl.gramene.org/Zea_mays/Info/Index). To identify the mutated genes, the genomic DNA of 25 candidate genes was amplified and sequenced. Sequence alignment revealed a single-nucleotide polymorphism in the E-subgroup PPR protein gene (Zm00001d014471). In the *dek55–1* mutant, nucleotide C was replaced with nucleotide T at + 449 bp, resulting in the substitution of the amino acid serine (Ser) with phenylalanine (Phe). However, no change in the mRNA expression level was observed (Fig. 2a-d). To validate our results, we obtained a new mutant, *dek55–2*, from the maize ethylmethanesulfonate-induced mutant database [34]. The *dek55–2* mutant showed a single-nucleotide mutation (G to A) at + 729 bp (Fig. 2b), which led to a truncated protein (Fig. 2d). The mutant *dek55–2* also produced defective kernels with a small white pericarp (Fig. 2e). An allelic test between *dek55–1* and *dek55–2* heterozygotes revealed that normal and mutant kernels segregated at the expected 3:1 ratio (normal/mutant; 450/143; *P* = 0.62) in the F_1_ ear (Fig. 2e). For a control, all the kernels from the ear that were crossed between the *dek55–2* heterozygote and WT were normal (Fig. 2e). Taken together, these results indicate that the mutation in the Zm00001d014471 PPR gene was responsible for the defective kernel phenotype, so the annotated gene was designated *DEK55*.

### DEK55 is a mitochondrial E-subgroup PPR protein

Sequence alignment demonstrated that the *DEK55* gene is a 1893 bp long ORF with no introns. Moreover, *DEK55* encodes a 630 amino acid protein containing 13 PPR motifs and an E domain at the carboxy-terminal end (Fig. 2b-d and Additional file 1: Fig. S1). Mutations in *dek55–1* and *dek55–2* were located in the third and fifth PPR motifs, respectively (Fig. 2d). The mutation in *dek55–2* resulted in a truncated DEK55 protein missing the last eight PPR motifs and the E domain.

**Fig. 3:**
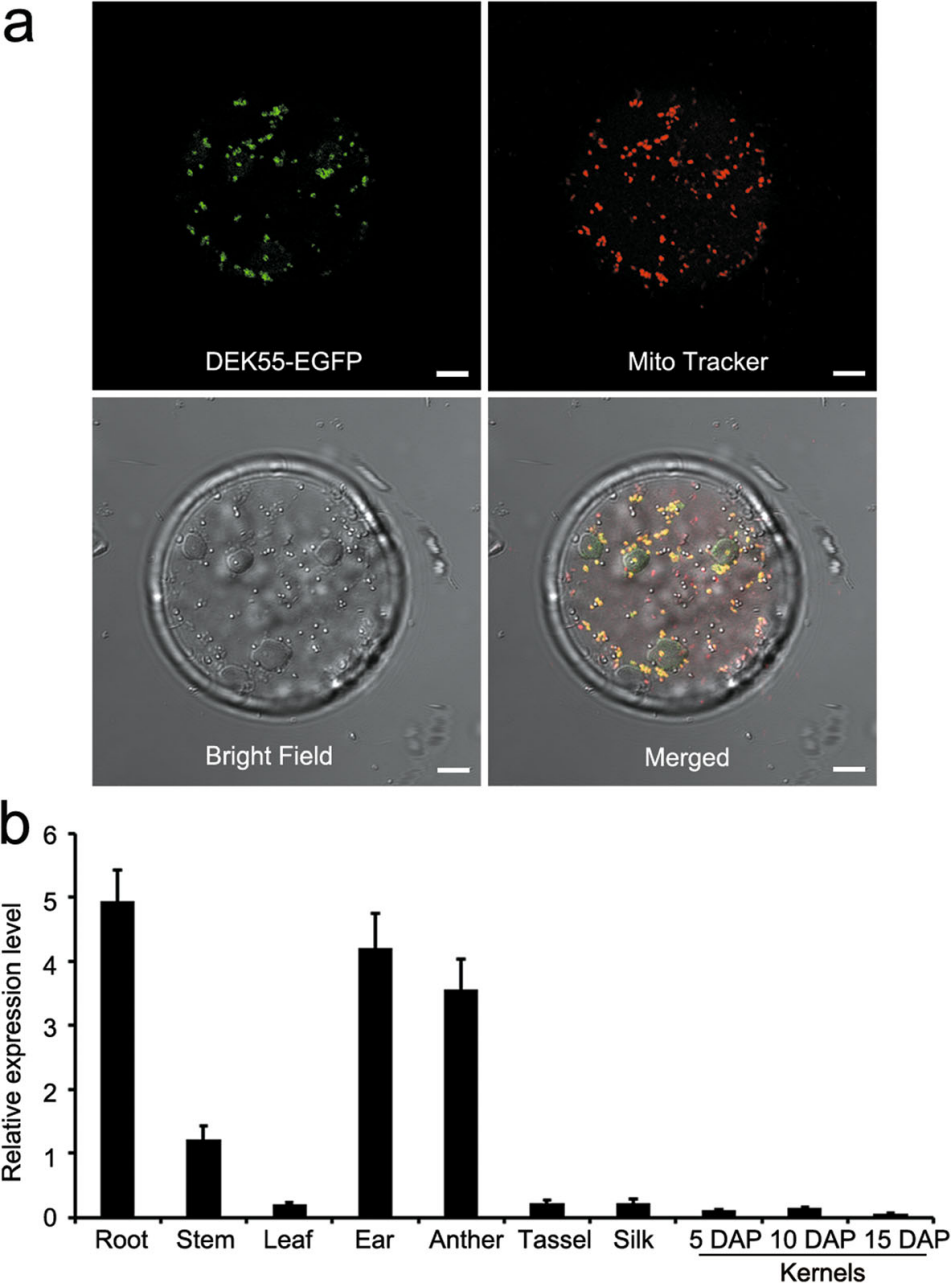
Subcellular localization of DEK55 and expression pattern of *DEK55*. **a** The subcellular localization of DEK55 was determined by transient expression of DEK55-EGFP fusion proteins in maize protoplasts. The mitochondria are stained by MitoTracker (red). Scale bars = 5 μm. **b** Analysis of the relative expression level of *DEK55* in various tissues and kernels at 5, 10, and 15 DAP. The *ZmActin* gene (GRMZM2G126010) was used as an internal control. The values are the means of three replicates. The error bars represent the standard deviations

To examine the subcellular localization of DEK55, a *p35S*:DEK55-enhanced green fluorescent protein (EGFP) vector was constructed and transformed into maize protoplasts. The fluorescent signal of DEK55-EGFP overlapped with that of MitoTracker (a mitochondrion-specific dye) (Fig. 3a), suggesting that, in maize, DEK55 is a mitochondrial PPR protein (Fig. 3a). In addition, expression analysis in various maize tissues demonstrated that *DEK55* is relatively highly expressed in the roots, anthers, and ears, with relatively low expression in the stems, leaves, silk, tassels, and kernels (Fig. 3b).

### DEK55 is involved in the C-to-U editing of 14 transcripts at multiple sites

**Fig. 4:**
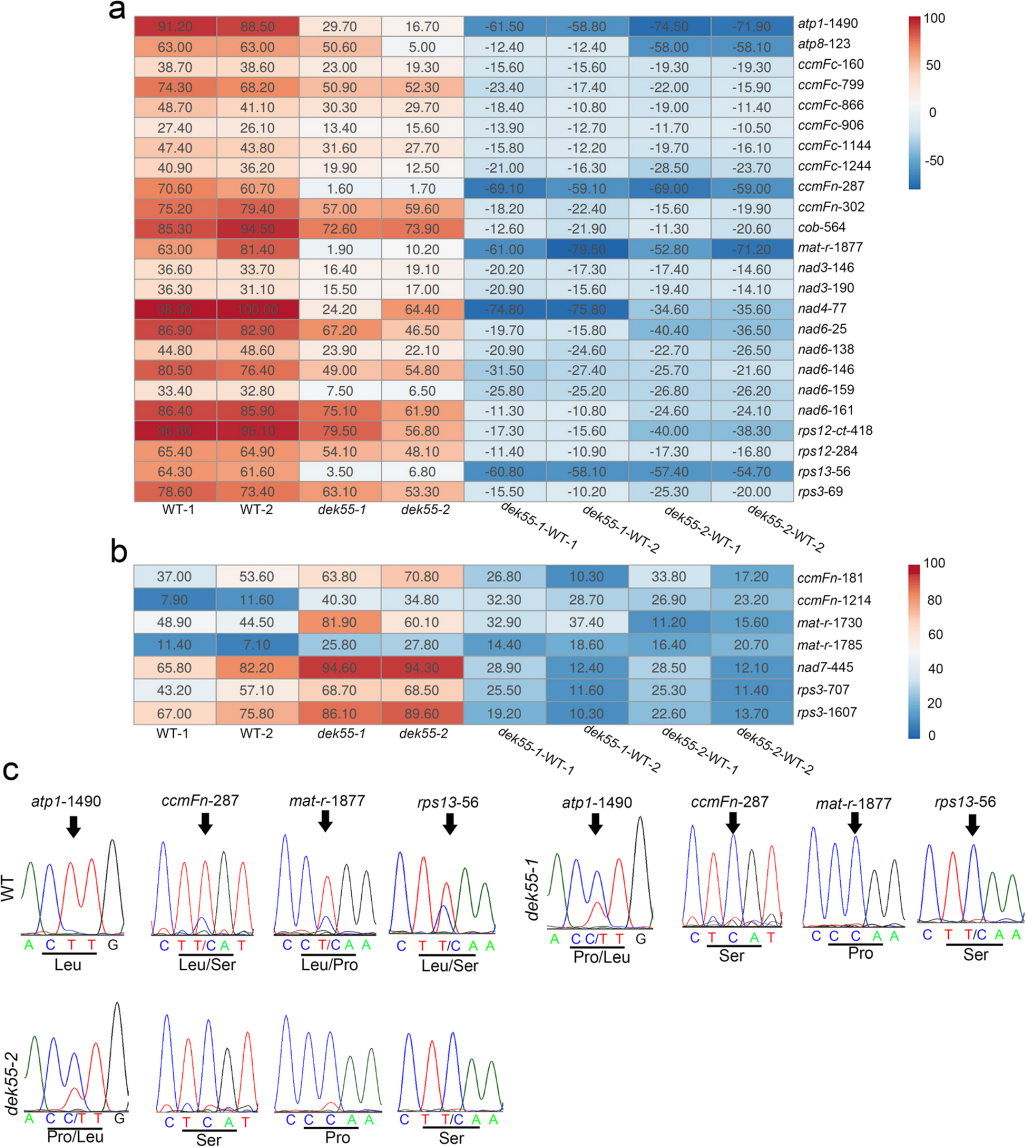
RNA C-to-U editing of 14 mitochondrial transcripts at multiple sites in maize mitochondria. **a-b** Heatmaps showing sites where the RNA editing efficiency in *dek55* decreased (**a**) and increased (**b**) compared to that in the WT. The editing efficiency of each site in the WT and *dek55* is indicated. The variation in editing efficiency of *dek55* compared to the WT is denoted by *dek55*-WT. **c** Sequence chromatograms containing the editing sites are shown. The arrows mark the editing sites. The amino acid in the editing site is indicated at the bottom

PPR proteins usually participate in modifying organelle transcripts [10]. It has been reported that E-subgroup PPRs are involved in the C-to-U editing of mitochondrial pre-mRNAs [14, 32, 33]. To explore whether DEK55 is involved in this processing, the transcript levels of 35 maize mitochondrial genes that encode functional proteins were analysed in both WT and *dek55–1* kernels. RNA editing of these transcripts in the *dek55* (*dek55–1* and *dek55–2*) and WT (WT-1 and WT-2) kernels was detected via the strand- and transcript-specific RNA sequencing (STS-PCRseq) strategy [35]. The sequencing reads were mapped to the 35 mitochondrial gene transcripts, and 482 C-to-U RNA editing sites were examined in the WT and *dek55* kernels (Additional file 2: Table S1). The results revealed that, compared with that of these RNA editing sites between the WT and *dek55* kernels (Additional file 2: Table S2), the C-to-U editing percentage of 31 editing sites in 14 transcripts (*atp1*, *atp8*, *ccmFc*, *ccmFn*, *cob*, *mat-r*, *nad3*, *nad4*, *nad6*, *nad7*, *rps12-ct*, *rps12*, *rps13*, and *rps3*) was significantly altered in the *dek55* kernels (Fig. 4, Additional file 2: Tables S2-S4), whereas the editing percentage of 24 sites decreased (Fig. 4a) and that of seven sites increased in the *dek55* kernels compared with WT kernels (Fig. 4b, Additional file 2: Tables S2 and S4). The editing efficiency at the *atp1*–1490, *ccmFn*-287, *mat-r*-1877, and *rps13*–56 sites dramatically decreased in the *dek55–1* and *dek55–2* kernels, and the editing percentage in the *dek55* mutants was more than 50% lower than that in the WT kernels (Fig. 4a, Additional file 2: Table S3). Direct sequencing of reverse transcription-polymerase chain reaction (RT-PCR) products of the *atp1*–1490, *ccmFn*-287, *mat-r*-1877 and *rps13*–56 sites also indicated that the editing efficiency was significantly reduced in the *dek55* kernels at these RNA editing sites (Fig. 4c). Deficient C-to-U RNA editing led to altered amino acid residues in *dek55* (Fig. 4c). Moreover, at the *atp8–*123 site, the editing efficiency of only the *dek55–2* kernels (5%) was more than 50% lower than that of the WT kernels, and at *nad4*–77 sites, the editing efficiency of only the *dek55–1* kernels (24.2%) was more than 50% lower than that of the WT kernels (Fig. 4a). Taken together, the above results indicated that DEK55 is required for RNA editing at multiple sites, especially the *atp1*–1490, *ccmFn*-287, *mat-r*-1877, and *rps13*–56 sites.

### DEK55 is essential for the *trans*-splicing of *nad1* introns 1 and 4 and the *cis*-splicing of *nad4* intron 1

**Fig. 5:**
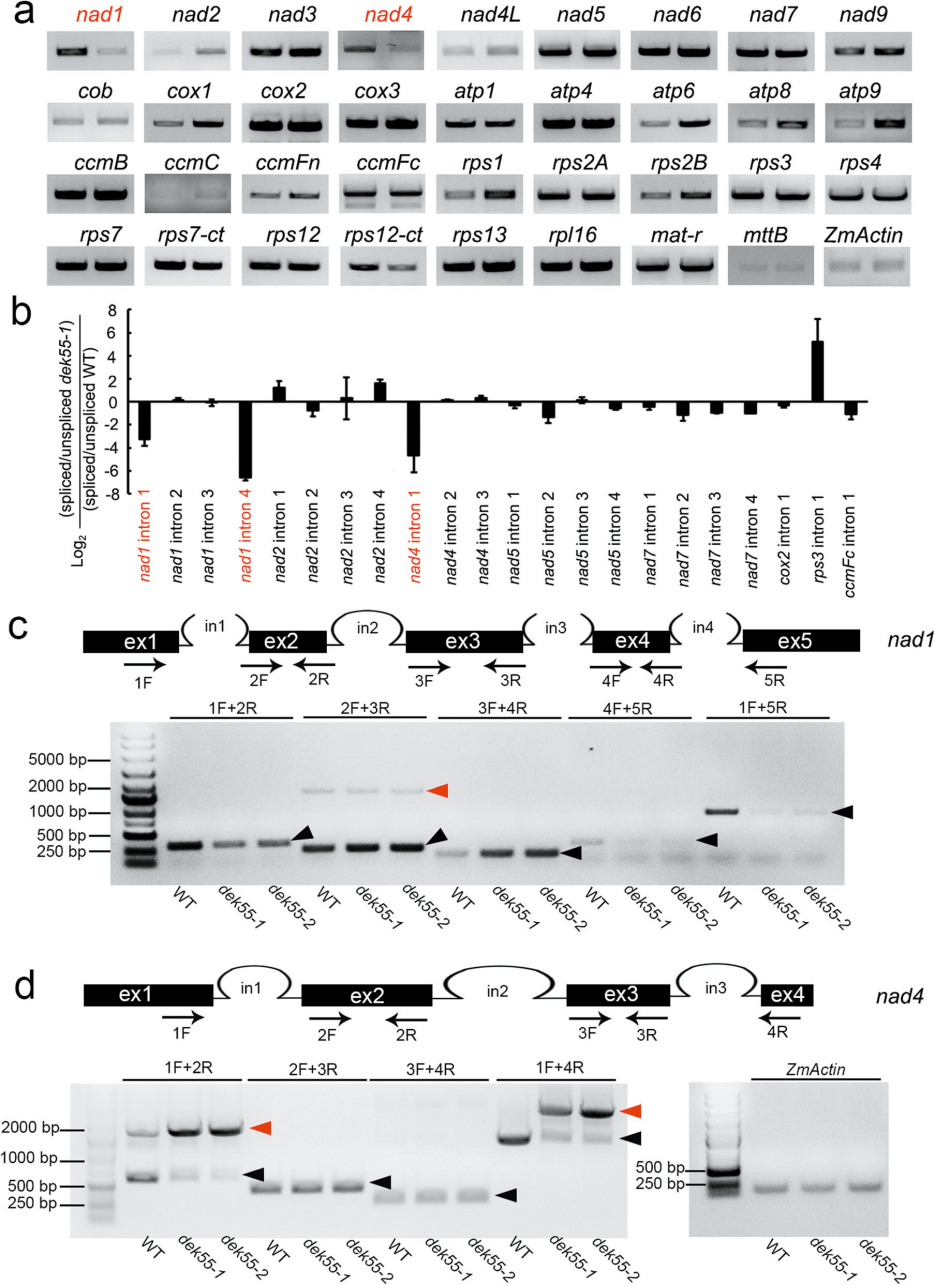
The posttranscriptional RNA processing of *nad1* and *nad4* was affected in *dek55*. **a** The expression of 35 mitochondrion-encoded genes in the WT (left) and *dek55–1* (right) kernels was detected via RT-PCR. The *ZmActin* gene (GRMZM2G126010) was used as an internal control. Both *nad1* and *nad4* are marked in red because their transcript abundance significantly decreased. **b** The splicing efficiency of all 22 group II introns in maize mitochondrial-encoded genes was determined in *dek55–1* and WT kernels via qRT-PCR. The values shown are the means of three biological replicates, and the error bars represent the standard deviations. **c-d** Schematic structure of the *nad1* gene (**c**) and *nad4* gene (**d**). The primers used for amplification are indicated. RT-PCR-based analysis of the intron splicing efficiency of *nad1* in WT kernels and in *dek55–1* and *dek55–2* mutant kernels at 15 DAP. All the PCR products were confirmed by sequencing. The *ZmActin* gene (GRMZM2G126010) was used as an internal control. The unspliced and spliced fragments are indicated by red and black arrowheads, respectively. Exons are indicated as “ex”, and introns are indicated as “in”. The gel images in (**a**, **c**, **d**) were cropped; the original gel images are shown in Additional file 1: Figs. S2-S3

The transcript levels of 35 maize mitochondrial genes were examined, and the results showed that the transcript levels of *nad1* and *nad4* were significantly downregulated in the *dek55* mutants (Fig. 5a). The genomic DNA of *nad1* contains four group II introns, and with the exception of the 2nd intron, all are *trans*-splicing introns. (Fig. 5c). The genomic DNA of *nad4* has three *cis*-splicing introns (Fig. 5d) [13, 36]. The full maturation of *nad1* and *nad4* transcripts requires complete intron splicing. We therefore further analysed the intron splicing efficiency of *nad1*, *nad4*, and other genes in the *dek55–1* and WT kernels via quantitative reverse transcription-polymerase chain reaction (qRT-PCR). Compared with that in the WT kernels, the splicing efficiency of the first and fourth introns of *nad1* and the first intron of *nad4* in the *dek55–1* mutant kernels decreased (Fig. 5b). Furthermore, we amplified each intron and full transcript of *nad1* and *nad4* via RT-PCR (Fig. 5c, d). The transcript abundance of *nad1* exons 1–2 and exons 4–5 and the full-length DNA fragments significantly decreased (Fig. 5c). RT-PCR could not amplify the intronic DNA fragments (1F + 2R, 3F + 4R, 4F + 5R) in the *dek55* and WT kernels because the 1st, 3rd, and 4th introns of *nad1* are *trans*-spliced. (Fig. 5c). The unspliced 2nd intronic fragments of *nad1* in the *dek55* mutant kernels were similar to those in the WT kernels (Fig. 5c). The abundance of *nad4* spliced exons 1–2 and full-length DNA fragments significantly decreased, and the abundance of *nad4* unspliced intron 1 transcripts significantly increased (Fig. 5d). Our findings suggest that the significant decrease in abundance of *nad4* and *nad1* transcript in the *dek55* mutant kernels was caused by the abnormal splicing of *nad4* intron 1, *nad1* intron 1, and intron 4 (Fig. 5a-d). Therefore, DEK55 is necessary for the *trans*-splicing of the two *nad1* introns (1st and 4th) and *cis*-splicing of the first *nad4* intron in maize.

### The *dek55–1* mutant exhibits reduced complex I activity and increased alternative respiratory pathway activity

**Fig. 6:**
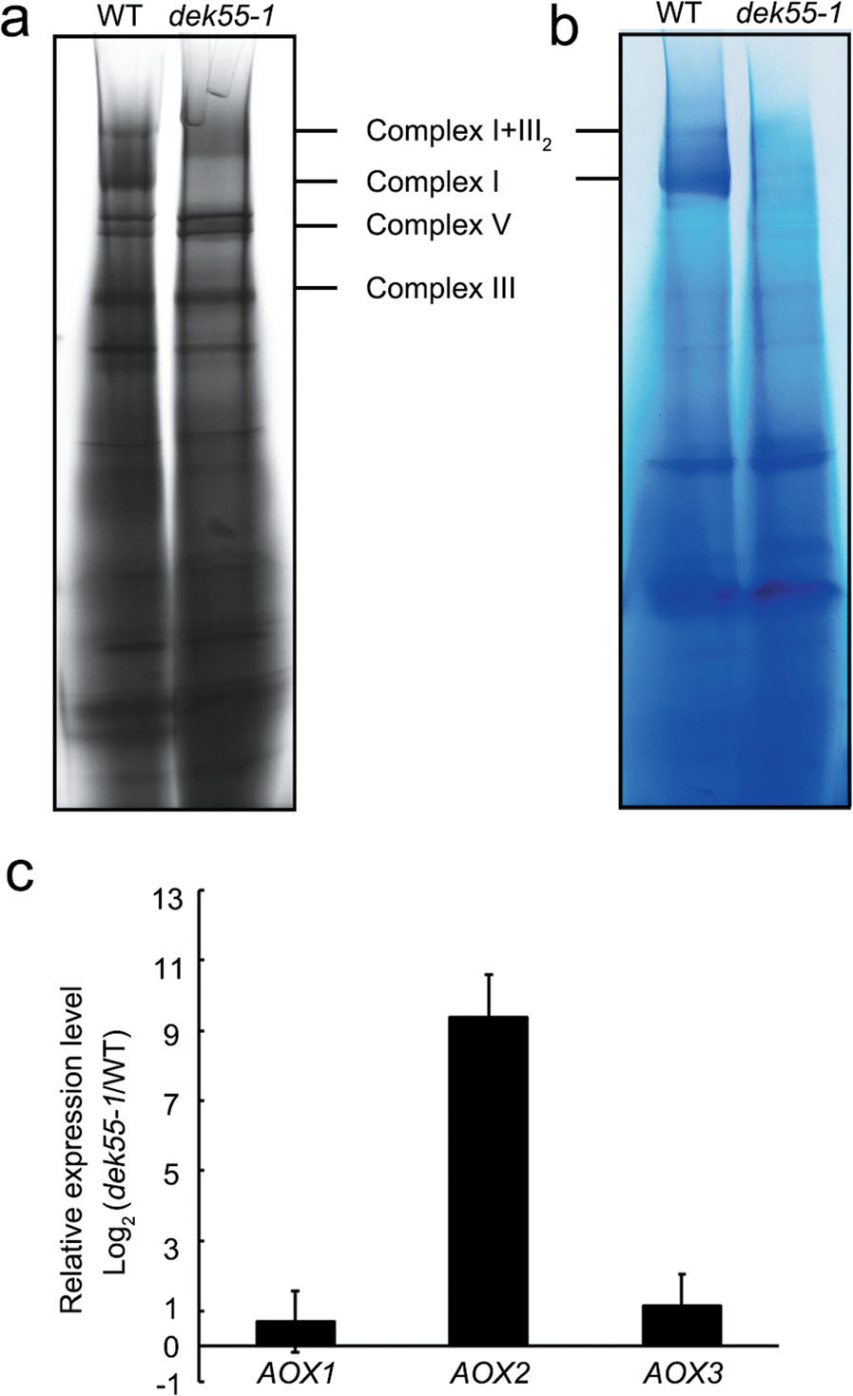
Mitochondrial function was impaired in the *dek55–1* mutant. **a** BN-PAGE analysis of mitochondrial complexes isolated from WT and *dek55–1* kernels at 15 DAP. The gels were stained with Coomassie brilliant blue. The positions of the mitochondrial complexes are marked. **b** In-gel NADH dehydrogenase activity analysis of complex I. The positions of complex I and supercomplex I + III_2_ are indicated. **c** qRT-PCR-based analysis of *AOX* gene (*AOX1*, *AOX2*, and *AOX3*) expression in WT and *dek55–1* kernels at 15 DAP. The *ZmActin* gene (GRMZM2G126010) was used as an internal control. The values shown are the means of three biological replicates, and the error bars represent the standard deviations. The gel images in (**a-b**) were cropped; the original gel images are shown in Additional file 1: Fig. S4

Four genes, i.e., *nad1*, *nad4*, *nad3*, and *nad6*, encode the subunits of complex I NAD1, NAD4, NAD3, and NAD6, respectively [36]. The *rps13* gene encodes a ribosomal protein, and *atp1* and *atp8* encode the ATPase subunit 1 and subunit 8 subunits of ATP synthase F1, respectively [36]. Defects in the posttranscriptional processing of these genes may impair the biosynthesis of mitochondrial complexes [17, 37–39]. We performed blue native polyacrylamide gel electrophoresis (BN-PAGE) and an in-gel NADH dehydrogenase activity assay to investigate the accumulation level and activity of mitochondrial complexes in WT and *dek55*–1 endosperm. BN-PAGE showed that the abundance of complex I and supercomplex I + III_2_ in the *dek55–1* mutant significantly decreased (Fig. 6a). However, no significant differences for complex V were observed between the WT endosperm and *dek55–1* endosperm (Fig. 6a). Furthermore, the activity of complexes I and I + III_2_ was reduced in the *dek55–1* mutant (Fig. 6b). Taken together, these results indicate that defects in mitochondrial transcript splicing and/or editing might affect the abundance and activity of mitochondrial complex I.

The mitochondrial respiratory chain in plants includes the cytochrome *c* and alternative oxidase (AOX) pathways [40]. When the main cytochrome *c* pathway is blocked, AOX activity can be increased to compensate for the respiratory pathway [41]. In *dek55–1*, the functions of complex I were abolished (Fig. 6a, b). Thus, we performed qRT-PCR to detect the expression levels of *AOX* genes in WT and *dek55–1* kernels, and the results showed a 512-fold increase in the expression level of the *AOX2* gene in the *dek55–1* mutant kernels compared to the WT kernels. (Fig. 6c). Collectively, our results indicate that the respiratory pathway is severely blocked in *dek55–1* mitochondria.

### DEK55 interacts with ZmMORF1 and ZmMORF8 in yeast

**Fig. 7:**
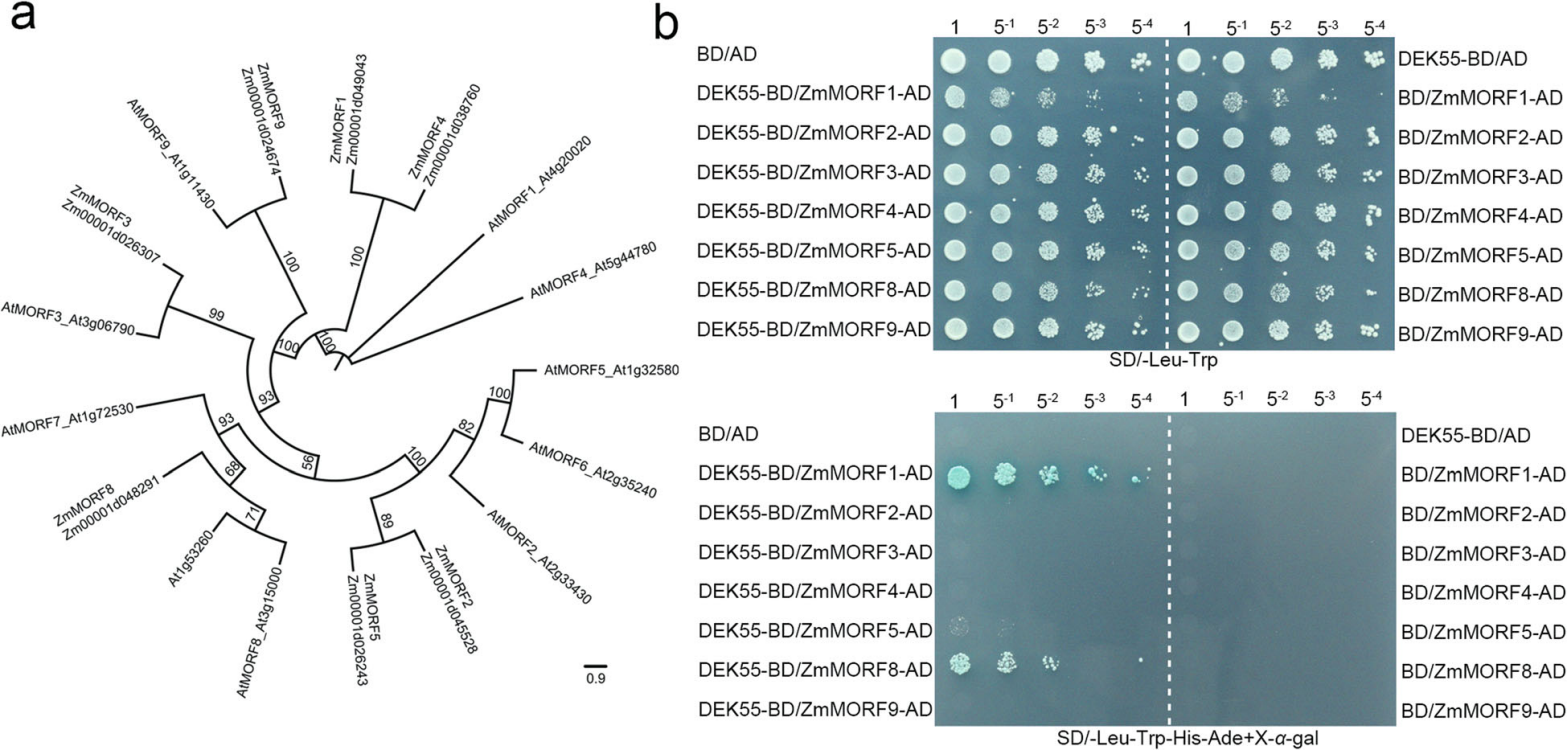
DEK55 interacts with ZmMORF1 and ZmMORF8 in yeast. **a** Phylogenetic tree of known AtMORFs in *Arabidopsis* and putative ZmMORFs in maize. **b** Yeast two-hybrid assay of the interaction between DEK55 and ZmMORFs. Yeast cells diluted to different concentrations (1, 5^− 1^, 5^− 2^, 5^− 3^, 5^− 4^) were cultured in SD/−Trp-Leu dropout and SD/−Trp-Leu-His-Ade plates supplemented with X-α-gal dropout media at 30 °C, and the results were recorded after 3 days of culture. The images in (**b**) were cropped; the original gel images are shown in Additional file 1: Fig. S5

Previous studies have shown that MORFs directly interact with PPR proteins and play a role in RNA editing at numerous editing sites [42, 43]. In the present study, DEK55 was found to be responsible for 31 RNA editing events in maize, so we speculated that DEK55 might interact with MORFs to form an editing complex involved in RNA editing in maize. Thus, we used MORFs in *Arabidopsis* as bait to search for putative MORFs in maize; seven putative MORFs were identified in maize (Fig. 7a). A yeast two-hybrid assay was performed to screen for MORFs that interact with DEK55, and the results indicated that DEK55 can interact with ZmMORF1 and ZmMORF8 in yeast (Fig. 7b).

## Discussion

### DEK55 is required for maize kernel development

Previous reports have shown that PPR proteins play vital roles in maize kernel development, and the loss of function of some PPR proteins results in empty pericarp and small and defective kernel phenotypes in different genetic backgrounds [13, 14, 17–21, 30, 31, 39, 44, 45]. The *ppr* mutants exhibit developmentally arrested embryos and endosperm. The embryos usually reached the coleoptile stage or leaf stage 1 (L1), and the endosperm presented significantly reduced starch and protein levels [14, 33, 45]. The *dek55* mutants produced small kernels with a shrivelled pericarp (Fig. 1a-c). Moreover, compared with the WT kernels, the mutant kernels had smaller embryos and a reduced proportion of hard endosperm (Fig. 1d-l). In particular, *dek55–1* embryos were severely arrested and had only one leaf primordium. Thus, these mutant kernels would not be able to germinate in the field. Allelic tests indicated that the nonsense mutant *dek55–2*, an allelic mutant with *dek55–1* and *dek55–1/dek55–2* heterozygous kernels, exhibited a phenotype similar to that of *dek55–1*. This suggests that *dek55* dysfunction is responsible for the defective kernel phenotype and that DEK55 is required for kernel development in maize.

The E-subgroup PPR proteins are characterized as having an E domain at their carboxy-terminal end, and this domain might be responsible for interactions between proteins [46, 47]. In the *dek55–1* mutant, there was a single-nucleotide change (C to T) at + 449 in the *dek55* gene, which resulted in Phe instead of Ser on the third PPR motif of DEK55 at position 150 in the polypeptide chain (Fig. 2b, d; Additional file 1: Fig. S1). This mutation from a hydrophilic amino acids to a hydrophobic amino acid would alter the affinity of the protein to water. Our evidence suggests that this amino acid change (Ser → Phe) is responsible for defective kernels in the *dek55* mutants. Therefore, the amino acid change at this site in DEK55 might cause conformational changes and loss of function. In the *dek55–2* mutant, the mutation resulted in a loss of the last eight PPR motifs and E domain at the carboxy-terminal end of the DEK55 protein (Fig. 2b, d), which might prevent it from forming complexes with other proteins and binding to its target sites.

### DEK55 is necessary for the C-to-U editing of multiple sites in the mitochondrial transcripts of maize

PPR proteins, including DYW2, EMP21, NUWA, MEF8, and DEK53, are involved in C-to-U editing at multiple sites [48–52]. In this study, we demonstrated that DEK55 is involved in RNA editing at 31 sites; however, the editing percentage of 24 editing sites and that of seven editing sites decreased and increased, respectively, in *dek55*, suggesting that DEK55 is also necessary for RNA editing at multiple sites. Among them, DYW2 and MEF8 harbour only five PPR repeats and belong to an atypical DYW subgroup [48, 49]. NUWA belongs to the P-class of PPR proteins [48, 50], and EMP21 contains 11 PPR motifs in addition to the E and DYW domains and belongs to the PPR-DYW protein family [51]. Furthermore, DEK53 is an E-subgroup PPR protein with seven PPR repeats [52], and DEK55 is considered an E-subgroup PPR protein that contains the canonical E domain. Therefore, PPR proteins that target multiple sites for editing might have dissimilar structures.

MORFs can interact directly with PPR proteins to participate in RNA editing [42, 43, 51]. In *Arabidopsis*, MEF13 (an E-subgroup PPR protein) interacts with MORF3 and MORF8; this protein complex is responsible for RNA editing of the same sites among *morf3*, *morf8*, and *mef13* mutants [42]. EMP21 is necessary for the editing of ~ 17% of mitochondrial target Cs in maize [51]. Interestingly, 34 editing sites overlap in maize *emp21* mutants and *Arabidopsis morf8* mutants, and eight editing sites overlap in maize *emp5* mutants and *morf8* mutants. ZmMORF8 (GRMZM2G169384), an orthologue of MORF8 in maize, directly interacts with EMP21 and EMP5, suggesting that EMP21 and EMP5 participate in the editing of several sites by interacting with ZmMORF8 [51]. DEK53 is an E-subgroup PPR protein that interacts with ZmMORF1 to form an RNA-editing complex and is responsible for more than 60 RNA edits in the maize mitochondrion [52]. In our study, DEK55 participated in the C-to-U editing of 14 mitochondrial transcripts at 31 editing sites, while the editing percentage of 24 sites decreased in *dek55* (Fig. 4a, Additional file 2: Table S3) (Fig. 4). Moreover, comparative analyses of these mitochondrial transcript C-to-U editing events in both *Arabidopsis* and maize indicated that multiple sites, e.g., *ccmFc*-799, *ccmFc*-866, *ccmFc*-1144, *ccmFc*-1244, *cob*-564, *mat-r*-1877, *nad3*–190, *nad4*–77, and *nad6*–146, were not edited in *Arabidopsis*, as these sites are “Ts”. This suggests that editing of these sites is necessary in maize. In addition, editing was also substantially impaired in *Arabidopsis morf8* mutants at the following four sites: *atp1*–1484 (*atp1*–1490 in the *dek55* mutants), *ccmFc*-160, *nad6*–161, and *rps12–*284 [35]. DEK55 can interact with ZmMORF8 (orthologue of AtMORF8 in maize), as shown by yeast two-hybrid assays. Although a *Zmmorf8* mutant has not yet been identified in maize, our findings might provide evidence that DEK55 might interact with ZmMORF8 to function at these RNA editing sites in maize. In *dek53*, more than 60 RNA editing sites were affected [52], and three of them (*nad3*–146, *nad6*–146, and *atp8*–123) were also affected in *dek55*. DEK53 and DEK55 interact with ZmMORF1 [52, this study]. Therefore, DEK53 and DEK55 might be responsible for C-to-U RNA editing of these sites by interacting with ZmMORF1. Taken together, these results indicate that ZmMORF8 and ZmMORF1 might interact with DEK55 to form an editing complex for these multiple editing sites.

### DEK55 is involved in group II intron splicing in maize mitochondria

E-subgroup PPR proteins are considered editing factors for RNA editing in organelles [10], but few of these proteins are considered to play a role in splicing [28, 29, 53]. SLO4 is necessary for RNA editing of *nad4* and the efficient splicing of *nad2* intron 1 in *Arabidopsis* mitochondria [29]. AEF1/MPR25 is involved in RNA editing of *atpF* and *nad5* and modulates *atpF* splicing in both *Arabidopsis* and rice [28]. Furthermore, the plastid PPR protein OTP70 participates only in the intron splicing of the *rpoC1* transcript [53]. In this study, DEK55 (an E-subgroup PPR protein) was shown to participate in both the RNA editing of 31 sites and group II intron splicing in maize mitochondrial transcripts (Figs. 4 and 5a-d). The RNA editing percentage of *nad1* transcripts was not affected, and among the *nad4* transcripts, only *nad4–77* transcripts decreased. Moreover, it has been reported that intron splicing can be mediated by RNA editing events in which the key sites of introns are edited [54–56]. The editing efficiency of two RNA editing sites (147 and 409) on *nad4* intron 1 increased in *dek55* (Additional file 2: Table S1), which might have resulted from the reduction in the splicing efficiency of *nad4* intron 1. Since splicing of *nad1* introns 1 and 4 occurs in trans, the introns were unable to be amplified via RT-PCR with primer pairs (1F + 2R and 4F + 5R) (Fig. 5c). The editing efficiency of the RNA editing sites on *nad1* introns 1 and 4 were not analysed. Thus, it could not be confirmed whether the decreased splicing efficiency of *nad1* introns 1 and 4 in *dek55* was caused by editing events of these introns.

Several proteins that participate in the splicing of *nad1* and *nad4* pre-mRNAs have been identified. Nuclear maturase 1 [57], DEK2 [45], and EMP11 [44] participate in the *trans*-splicing of *nad1* intron 1, and EMP11, EMP8, and ZmSMK3 are required for *nad1* intron 4 *trans*-splicing [44]. The proteins NMS1 [58], DEK35 [19], EMP8 [13], DEK43 [20], EMP602 [59], and ZmSMK3 [60] and are involved in *cis*-splicing of *nad4* intron 1. The results of our study demonstrated that DEK55 is involved in both *trans-* and *cis-*splicing. It appears that splicing of one intron may require the involvement of multiple factors to constitute a putative spliceosome. This is supported by the finding that PPR-small MutS-related-1 can interact with Zm-mCSF1 to form a protein complex. This protein complex is subsequently involved in the intron splicing of multiple transcripts within the mitochondria [61]. Therefore, DEK55 might interact with P-type PPR proteins or other splicing factors involved in group II intron splicing.

## Conclusions

In this study, we demonstrated that DEK55 is a mitochondrion-localized E-subgroup PPR protein. Mutation of *DEK55* leads to embryo lethality and arrested endosperm development in maize. DEK55 is required for editing at 31 RNA editing sites, especially the *atp1*–1490, *ccmFn*-287, *mat-r*-1877, and *rps13*–56 sites (Fig. 4). Moreover, DEK55 can interact with ZmMORF1 and ZmMORF8 in yeast. In addition, DEK55 is responsible for the *trans*-splicing of two *nad1* introns (intron 1 and intron 4) and the *cis*-splicing of *nad4* intron 1 in the mitochondria. Our results suggest that the E-subgroup PPR protein DEK55 plays important roles in the RNA editing and splicing of introns of maize mitochondrial transcripts. These results provide a novel perspective for understanding the molecular function of E-subgroup PPR proteins in RNA processing in plant organelles.

## Methods

### Plant materials

The maize mutant *dek55–1* identified from an ethylmethanesulfonate population in the B73 background was kindly provided by Prof. Xiaoduo Lu of Qilu Normal University. The original name of the allele mutant *dek55–2* was EMS4–073342, which was purchased from a maize ethylmethanesulfonate-induced mutant database (http://www.elabcaas.cn/memd/) [34] and identified by searching for the gene ID (Zm00001d014471). To purify the genetic background, *dek55–1* was back-crossed with the B73 inbred line, and BC_2_F_2_ kernels were used in this study. A *dek55–1* heterozygote (as the male parent) was crossed with our laboratory-grown inbred lines C733 and S162, after which the F_1_ progeny were self-pollinated to generate an F_2_ population that was used for gene mapping. Ru Chang Ren and Xu Wei Yan formally identified the plant materials. All the plant materials were sown at the experimental station of Shandong Agricultural University (Taian, Shandong Province).

### Histological analysis

WT and defective kernels were obtained from self-pollinated heterozygous plants at 12 and 18 DAP, respectively. The middle part of the kernel along the longitudinal axis was selected and placed in formalin-acetic acid-alcohol solution for at least 12 h on ice, followed by treatment with 50, 70, 85, 95, and 100% ethanol. Afterwards, the seeds were placed in 100% xylene for 2–4 h. After dehydration, the materials were immersed in molten paraffin for 72 h at 60 °C and then embedded in the paraffin. The paraffin-embedded samples were cut into 12 μm slices using a microtome (Leica RM2235, Germany). The sections were then stained based on the methods of Ren et al. [20]. The sections were ultimately imaged with a light microscope equipped with a camera (Olympus DP72, Olympus, Tokyo, Japan).

### Map-based cloning

The *DEK55* locus was identified using 1868 F_2_ defective kernels from the self-pollinated F_1_ population (C733 × *dek55–1/+*). For preliminary mapping, 73 polymorphic SSR markers selected from the entire genome were used to screen the DNA of the parents, individual F_1_ plants, and four groups of pooled F_2_ defective kernels. For fine mapping, new molecular markers were selected according to the parental DNA sequences. A website (http://ensembl.gramene.org/Zea_mays/Info/Index) was used to search for the genes annotated in the candidate regions of the *Zea mays* genome (B73_RefGen_v4) [62]. Phanta EVO Super-Fidelity DNA polymerase (catalogue number P503-d1, Vazyme Biotech Co., Nanjing, China) was used to clone all the candidate gene genomic DNA sequences and sequencing. The primers used were designed according to the candidate gene reference sequences. The primers used to clone the full-length *DEK55* gene and used for map-based cloning are given in Additional file 1: Table S2.

### RNA extraction, RT-PCR, and qRT-PCR

Total RNA from WT and *dek55* mutant kernels without a pericarp and other tissues was extracted with an Ultrapure RNA Kit (CWBIO, China). Any residual DNA among the total RNA was removed by DNase. For RT-PCR, complementary DNA (cDNA) was obtained by reverse transcription and used as the template for polymerase chain reaction (PCR)-based amplification. KOD DNA polymerase (KOD FX Neo, code: KFX-201, Toyobo, Japan) was used for PCR. The PCR procedure was as follows: initial melting at 94 °C for 2 min; 33 to 38 cycles of 15 s at 98 °C and 30 s at the applicable annealing temperature (58 °C to 61 °C) for the various primer pairs; adequate extension (30 s/kb) at 68 °C; and a final extension at 68 °C for 7 min. RT-PCR was performed to amplify the mitochondrial transcripts, splicing efficiency of *nad1* and *nad4* introns. The DNA fragments obtained via RT-PCR were directly sequenced. The transcripts were amplified by the use of previously reported primers [63], which are listed in Additional file 1: Table S2. The primers used to amplify the introns of *nad1* and *nad4* are shown in Additional file 1: Table S2.

The qRT-PCR equipment and reaction system were the same as those of a previous report [20]. All the qRT-PCR assays were performed for three samples and with three technical repeats. The primers for group II intron splicing efficiency analysis in the mitochondria were designed according to previous reports [17, 18, 63]. The primers used to analyse *DEK55* expression levels are shown in Additional file 1: Table S2.

### RNA editing efficiency analysis

The RNA editing of 35 mitochondrial genes was analysed via the STS-PCRseq method described by Bentolila et al. [35], with slight modifications. The 35 mitochondrial gene transcripts and *nad4* intron 1 were amplified through RT-PCR using four cDNA libraries as templates. These cDNA libraries were obtained from WT and *dek55* mutant kernels (WT-1 and *dek55–1*; WT-2 and *dek55–2*) obtained from self-pollinated *dek55–1/+* and *dek55–2/+* heterozygous ears at 15 DAP. The PCR products were visualized on 1% agarose gels; the gel bands were excised, and the DNA fragments were purified using a Gel DNA Extraction Mini Kit (catalogue number DC301–01, Vazyme Biotech Co.,). Purified DNA samples amplified from the same cDNA library were mixed together in equimolar amounts and sonicated to generate 300–500 bp DNA fragments. The DNA library was constructed and sequenced on the Illumina platform by Novogene Bioinformatics Technology Co., Ltd. (Beijing, China). The sequenced data were filtered to remove any low-quality reads, adaptor reads, and unknown base reads. The clean reads were subsequently mapped to the 35 mitochondrial gene transcripts and *nad4* intron 1 using Bowtie 2, and allele frequency counts were performed as described previously [52]. The editing efficiencies of 482 RNA editing sites were calculated, and the affected editing site in the *dek55* mutants was defined as described previously [51]. Editing efficiency was considered to have decreased in the *dek55* mutants when the T/(T + C)% in *dek55*-T/(T + C)% in the WT was ≤ − 10%, while the editing efficiency was considered to have increased in the *dek55* mutants when the T/(T + C)% in *dek55*-T/(T + C)% in the WT was ≥10%. Overlapping sites affected in *dek55–1* vs. WT-1, *dek55–1* vs. WT-2, *dek55–2* vs. WT-1, and *dek55–2* vs. WT-2 were considered affected editing sites [51].

### Yeast two-hybrid assays

The full-length ORF of DEK55, excluding the signal peptide coding sequence (1–207 bp), was amplified using specific primers. The PCR products were subsequently cloned into a pGBKT7 vector (Clontech, Kyoto, Japan) at the *EcoRI* and *BamHI* sites to generate a DEK55-BD bait vector. The coding sequences of seven ZmMORFs were then amplified using specific primers. The PCR products were subsequently cloned into the pGADT7 vectors (Clontech) to generate recombinant ZmMORF-AD prey vectors. The DEK55-BD and ZmMORF-AD recombinant vectors were cotransfected into Y2HGold competent cell (catalogue number YC1002, Weidi Biotechnology Co., Shanghai, China). Empty pGBKT7 and pGADT7 vectors were used as negative controls. The transformed cells were incubated on synthetic dextrose (SD)/−leucine (Leu)-Trp dropout plates and SD/−Leu-Trp-His-Ade dropout plates supplemented with X-*α*-gal at 30 °C for 3 days. The primers used are listed in Additional file 1: Table S2.

### Subcellular localization

The complete ORF (excluding the stop codon) of the *DEK55* gene was incorporated into a pM999-EGFP vector, generating a DEK55-EGFP recombinant vector driven by the CaMV 35S promoter. Subcellular localization was performed as reported previously [64]. In brief, maize mesophyll cell protoplasts were obtained from etiolated leaves by enzymatic hydrolysis as described previously [21]. Recombinant vectors (20 μL, 15–20 μg) were added to a 200 μL maize protoplast solution, and 220 μL of 40% (w/v) PEG4000 solution was then added and mixed completely, after which the samples were incubated at 23 °C for 10–15 min. Afterwards, the protoplasts were washed using a W5 or WI solution and cultured for 12–16 h in the dark at 23 °C. Before being imaged, the protoplasts were stained with a mitochondrion-specific dye (MitoTracker Red CMXRos, Thermo Fisher Scientific, Waltham, MA, USA), and the samples were observed using a laser confocal microscope (LSM 880, Zeiss, Jena, Germany).

### Isolation and analysis of mitochondrial complexes

A plant mitochondrial isolation kit (catalogue number P0045, Biohao, Wuhan, China) was used to isolate crude mitochondria from WT and *dek55–1* seed tissue, excluding the pericarp (at 15 DAP), for BN-PAGE and complex I activity analysis. The collected mitochondrial precipitate was redissolved in 35 μL of solution buffer (50 mmol/L bis-Tris, 6 N HCl, 50 mmol/L NaCl, 10% w/v glycerol, 0.001% Ponceau S; pH 7.2) containing 20% n-dodecyl-b-D-maltoside (Sigma-Aldrich, St. Louis, MO, USA) to a final concentration of 1%) and then kept on ice for 30 min. The suspension was then centrifuged at 4 °C, after which the supernatant was collected and loaded on preprepared gradient gels (BN1002BOX, Thermo Fisher Scientific), and electrophoresis was performed according to the manufacturer’s instructions. Afterwards, the gels were placed in 100 mL of fixing solution (methanol:ddH_2_O:acetic acid, 4:5:1) for 30 min and then transferred to 0.02% Coomassie R-250 stain (Sigma-Aldrich, St. Louis, MO, USA) for mitochondrial complex abundance analysis. The gel strips were incubated in assay buffer (25 mg nitro blue tetrazolium and 100 μL of NADH (10 mg/mL) combined with 10 mL of 5 mmol/L Tris/HCl; pH 7.4) (Sigma-Aldrich) for 5 min, and the reaction was terminated with the fixing solution (40% methanol:10% acetic acid (v/v)) for analysis of complex I activity [44].

## Supplementary Information

**Additional file 1: Fig. S1.** Amino acid alignment of maize DEK55 with homologous PPR proteins of other plant species. **Fig. S2.** Original gel images corresponding to Fig. 5a. **Fig. S3.** Original gel images corresponding Fig. 5c-d. **Fig. S4.** Original gel images corresponding to Fig. 6a-b. **Fig. S5.** Original gel images corresponding to Fig. 7b. **Table S1.** Genetic analysis of the mutant kernels in the segregating ear. **Table S2.** Primers used in this study.

**Additional file 2: Table S1.** Number of reads at each editing site for each library. **Table S2.** Editing percentage at each editing site of each library. **Table S3.** The editing percentage of 24 RNA editing sites decreased in the *dek55* mutant kernels. **Table S4.** The editing percentage of seven RNA editing sites increased in the *dek55* mutant kernels.

## Data Availability

All data generated or analysed during this study are included in this published article and its supplementary information files. The datasets used and/or analysed during the current study are available from the corresponding author on reasonable request. Sequencing data for RNA editing efficiency analysis has been deposited in National Center for Biotechnology Information Sequence Read Archive database (https://trace.ncbi.nlm.nih.gov/Traces/sra/), the BioProject accession number: PRJNA679100.

## Abbreviations

AOX: Alternative oxidase
atp1: ATP synthase subunit 1
BN-PAGE: Blue native polyacrylamide gel electrophoresis
C: Cytidine
cox: Cytochrome c oxidase
cDNA: Complementary DNA
DAP: Days after pollination
dek: Defective kernel
EGFP: Enhanced green fluorescent protein
EMP: Empty pericarp
Leu: Leucine
MEF: Mitochondrial editing factor
MORF: Multiple organellar RNA-editing factor
MPR25: Mitochondrial PPR 25
ORF: Open reading frame
OTP87: Organelle transcript processing 87
PCR: Polymerase chain reaction
Phe: Phenylalanine
PPR: Pentatricopeptide repeat
Pro: Proline
qRT-PCR: Quantitative reverse transcription-polymerase chain reaction
rps13: Ribosomal protein S13
RT-PCR: Reverse transcription-polymerase chain reaction
SD: Synthetic dextrose
Ser: Serine
SLO1: Slow growth 1
SMK: Small kernel
SSR: Simple sequence repeat
STS-PCRseq: Strand- and transcript-specific RNA sequencing
U: Uridine
WT: Wild type
