# Supplementary material for: The novel E-subgroup pentatricopeptide repeat protein DEK55 is responsible for RNA editing at multiple sites and for the splicing of nad1 and nad4 in maize
Source: BMC Plant Biol. 2020 Dec 9;20:553. doi: 10.1186/s12870-020-02765-x (PMC7727260; doi:10.1186/s12870-020-02765-x)
Supplement: Supplementary file 1 — Additional file 1: Fig. S1. Amino acid alignment of maize DEK55 with homologous PPR proteins of other plant species. Fig. S2. Original gel images corresponding to Fig. 5a. Fig. S3. Original gel images corresponding Fig. 5c-d. Fig. S4. Original gel images corresponding to Fig. 6a-b. Fig. S5. Original gel images corresponding to Fig. 7b. Table S1. Genetic analysis of the mutant kernels in the segregating ear. Table S2. Primers used in this study. [file 12870_2020_2765_MOESM1_ESM.docx]

**Additional file 1**

**Supplementary figure**

**
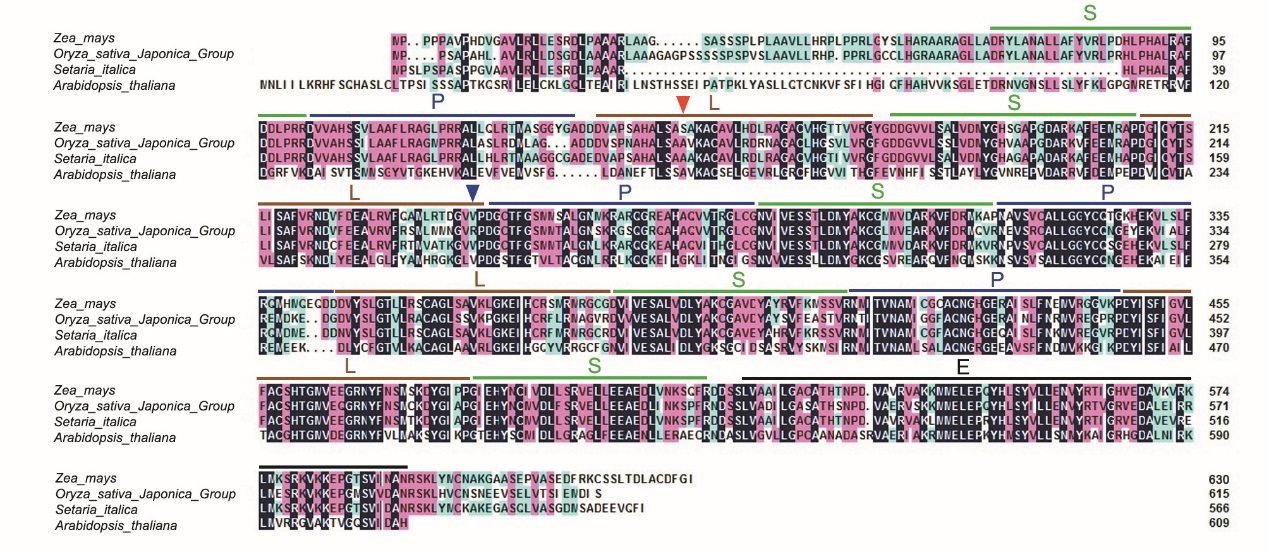
**

**Fig. S1. Amino acid alignment of maize DEK55 with homologous PPR proteins of other plant species.**

PPR elements P, L and S are indicated by a green, blue and red bar, respectively. The mutant sites in *dek55-1* and *dek55-2* are indicated by red and blue arrow heads, respectively. E domain is indicated by black bar. *Arabidopsis thaliana*, sequence ID NP_171853.1; *Oryza sativa Japonica Group*, sequence ID XP_015643919.1; *Setaria italica*, sequence ID XP_022681874.1. All data obtained from the NCBI (<https://www.ncbi.nlm.nih.gov/)>

_
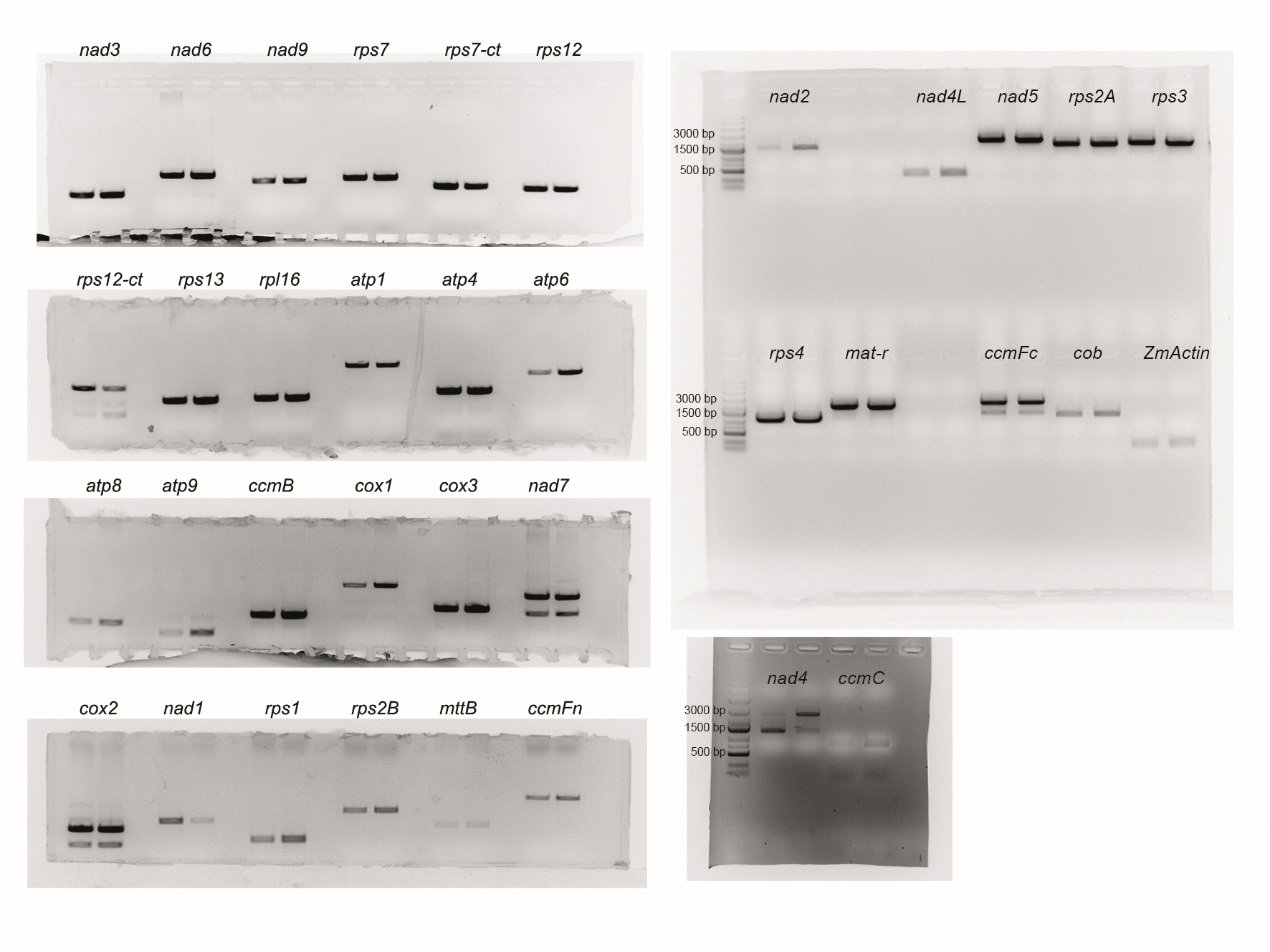
_

**Fig. S2. Original gel images corresponding to Fig. 5a.**

The RT-PCR products were detected by 1.0% agarose gel electrophoresis. The genes name were marked.


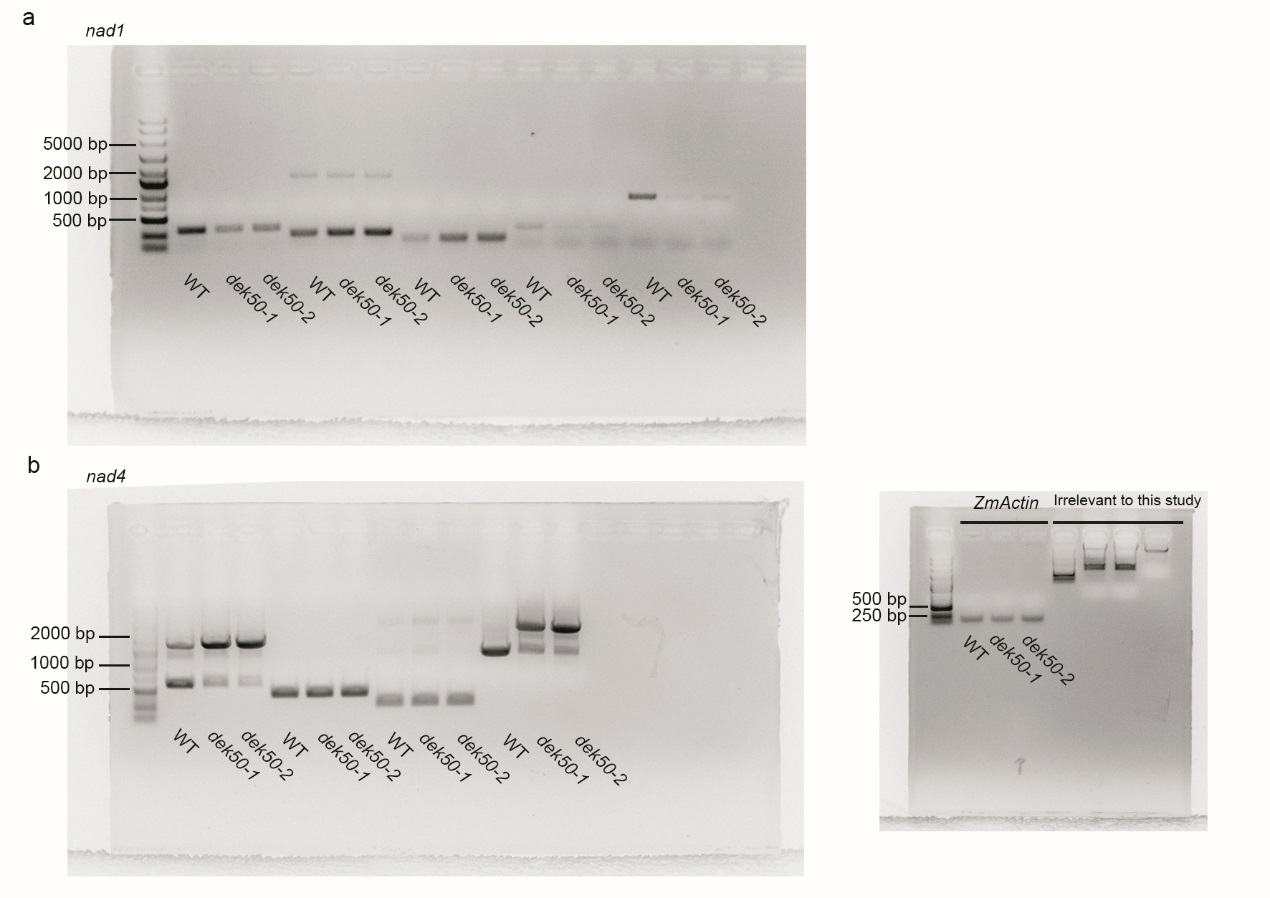


**Fig. S3. Original gel images corresponding Fig. 5c-d.**

**(a)** Original gel images of RT-PCR analysis of intron-splicing efficiency of *nad1* in WT, *dek55-1* and *dek55-2* mutant kernels.

**(b)** Original gel images of RT-PCR analysis of intron-splicing efficiency of *nad4* in WT, *dek55-1* and *dek55-2* mutant kernels. *ZmActin* gene (GRMZM2G126010) was used as an internal control and original gel image was also shown. Results irrelevant to this study were marked with “Irrelevant to this study”.


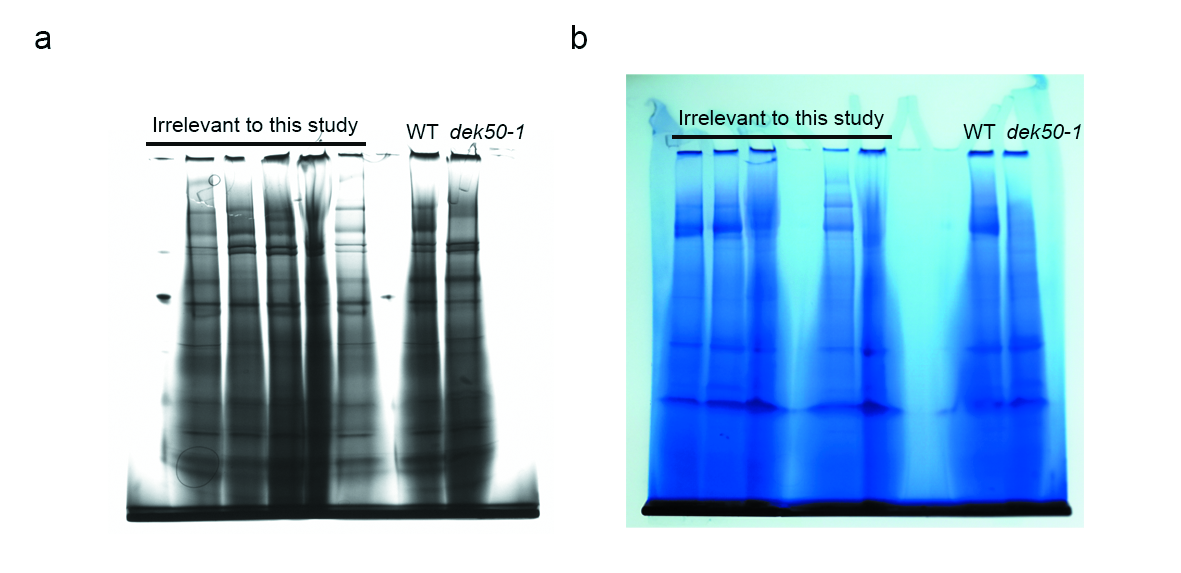


**Fig. S4. Original gel images corresponding to Fig. 6a-b.**

**(a)** Original gel images of BN-PAGE analysis of mitochondrial complexes.

**(b)** Original gel images of in-gel NADH dehydrogenase activity analysis of complex I.

Results irrelevant to this study were marked with “Irrelevant to this study”.


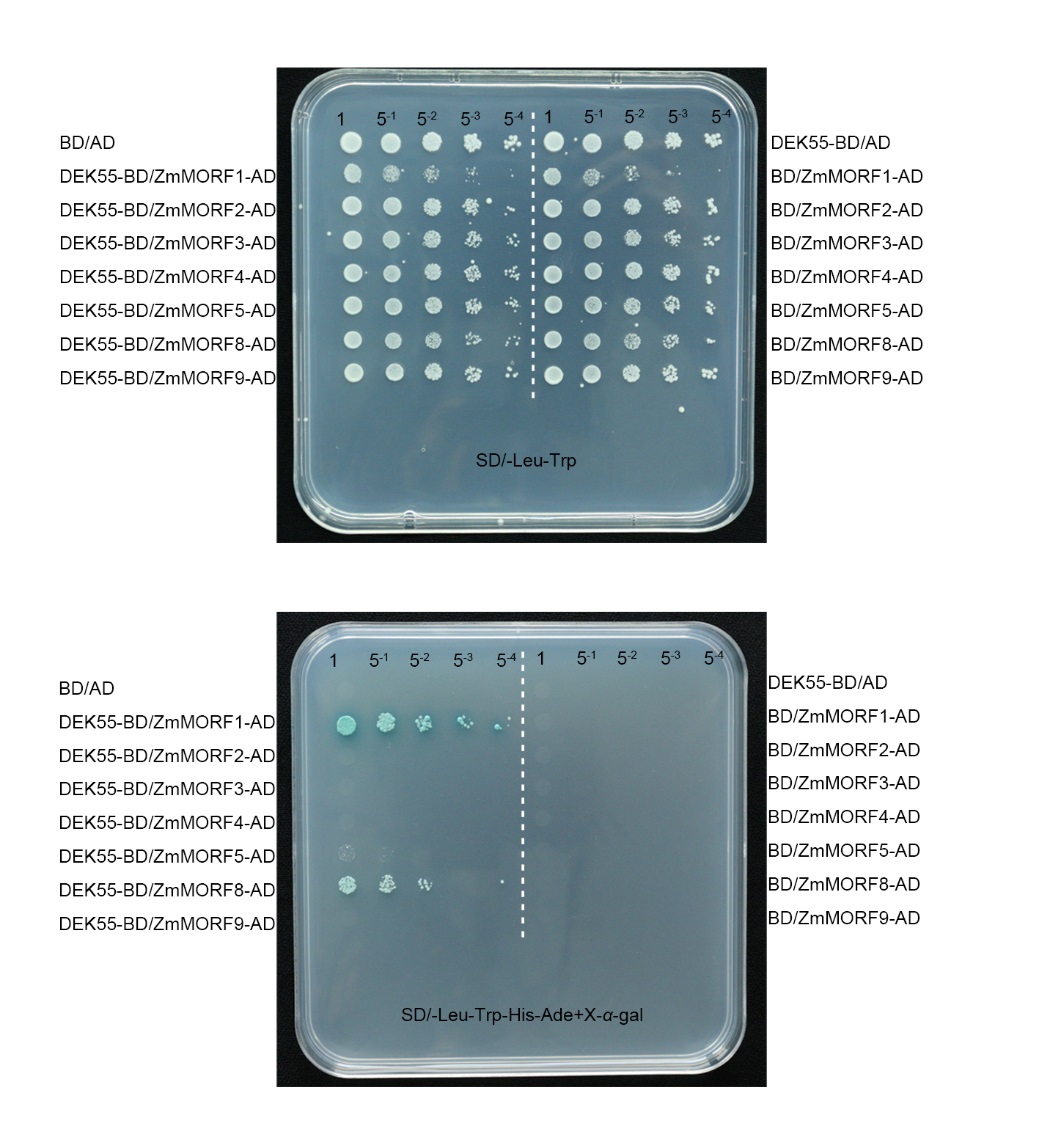


**Fig. S5. Original gel images corresponding to Fig. 7b.**

**Supplementary Table**

**Table S1. Genetic analysis of the mutant kernels in the segregating ear.**

| Self-pollinated parents | Number of mutants | Number of WT | Actual ratio | Expected ratio | *P* |
| --- | --- | --- | --- | --- | --- |
| *dek55-1/+* | 411 | 1239 | 1:3.01 | 1:3 | 0.93 |
| F_1_ (C733×*dek55-1/+*) | 916 | 2821 | 1:3.07 | 1:3 | 0.49 |
| F_1_ (S162×*dek55-1/+*) | 457 | 1364 | 1:2.98 | 1:3 | 0.92 |

**Table S2. Primers used in this study.**

| umc1705-F | ATCTCACGTACGGTAATGCAGACA | For fine mapping |
| --- | --- | --- |
| umc1705-R | CATGACCTGATAAACCCTCCTCTC |  |
| umc2302-F | GCATATGCGAGATCATATCGTTGA |  |
| umc2302-R | CTATACAGCCCTCAGCTCTGCTGT |  |
| M2-F | GTGCTATGCATGAGGGTAGAGT |  |
| M2-R | TGTATTGGGCGATACCGAGC |  |
| M3-F | ATCTGTAGTGCTTCCGTGCTCT |  |
| M3-R | GGCTCATTTGTAAGGTTGTGC |  |
| M4-F | GTTGAGATAACTATGCAAATCGTG |  |
| M4-R | CTTCCAACGTGCGTCTCTAC |  |
| M5-F | CGTCGTCGAGGTGATTAGGG |  |
| M5-R | GAACCCTCCTCCCGACTACT |  |
| M6-F | GGTTTCCAGAGCTAGGTGGG |  |
| M6-R | CGTCGTTTCCGTCGTCAATC |  |
| F148-F | GTTCGGTCGAGTTGGGATGA |  |
| F148-R | CAGACCAACCCTTCAGTCCC |  |
| DEK55-Full-F | GCCAGGTAGAATAGATGGAACC | Clone *DEK55* full length |
| DEK55-Full-R | CAAGAGCTGCTAAAATTTGTCAGTAC |  |
| ZmActin-F | ATGGTCAAGGCCGGTTTCG | qRT-PCR and RT-PCR primer of *ZmActin* |
| ZmActin-R | TCAGGATGCCTCTCTTGGCC |  |
| DEK55-qRT-F | CGATGACTGGTATAGCTTAGGG | qRT-PCR analysis of *DEK55* gene expression |
| DEK55-qRT-R | CGCATCCTCTCATCCTCATAG |  |
| nad1-1F | GCAACGTAGAAAGGGTCCTG | Test *nad1* introns splicing efficiency |
| nad1-2R | TGAGCTGCAGATCGTAATGC |  |
| nad1-2F | TCGAAATATGCCTTTCTAGGAG |  |
| nad1-3R | ATTCAGCTTCCGCTTCTGG |  |
| nad1-3F | GTCATGGCGCAAAAGCAGATATGG |  |
| nad1-4R | AGAGCAGACCCCATTGAAGA |  |
| nad1-4F | TCTTCAATGGGGTCTGCTCT |  |
| nad1-5R | AGGGAGCCATCGAAAGGTGA |  |
| nad4-1F | TTGATTGGTCTGTGCGTTTC | Test *nad4* introns splicing efficiency |
| nad4-2R | GGCTTCGGGTAACCAAATA |  |
| nad4-2F | TGCCGTCAAAGTGCCTAT |  |
| nad4-3R | GGTGCTCACTAAACCTCCATA |  |
| nad4-3F | CACTTTAGCCAATATGAGTTTACC |  |
| nad4-4R | ATGCAGTCCGGGAACACT |  |
| AOX1-qRT-F | AAGGTGCTGCTCGACAAGAT | qRT-PCR analysis of *AOX1* gene expression |
| AOX1-qRT-R | GTAGGCGTTGAAGAAGACGC |  |
| AOX2-qRT-F | GACATCTTCTTCCAGAGGCG | qRT-PCR analysis of *AOX2* gene expression |
| AOX2-qRT-R | TGACTACGTCCTTGAGCGTG |  |
| AOX3-qRT-F | CCAAGCTCGTGAAGGAAGAC | qRT-PCR analysis of *AOX3* gene expression |
| AOX3-qRT-R | GTAGGCGTTGAAGAAGACGC |  |
| DEK55-SC-F | CCGCGAATTCATGCCGCCGCCCCCAGCCG | Construct full length *DEK55-EGFP* vector |
| DEK55-SC-R | CCGCGGTACCGATACCAAAATCACAAGC |  |
| nad4-F1 | TTGATTGGTCTGTGCGTTTC | RT-PCR |
| nad4-R1 | ATGCAGTCCGGGAACACT |  |
| nad4-F | CAGTCACCCGGAGAAGATTT | Gene sequencing in RNA editing efficiency analysis |
| nad4-R | TAATTTGGCGCCTGATTGAC |  |
| nad2-F | GACGGAGGAGAGGAAATGAA | RT-PCR, Gene sequencing in RNA editing efficiency analysis |
| nad2-R | GCAGTCCACCCTTTCTTTGA |  |
| nad3-F | CTTTCCTATGTCCTTCCCCC |  |
| nad3-R | GAGGAGAGCGAGAGAACGAA |  |
| nad4L-F | CTGACATTCCATGTTTCCGA |  |
| nad4L-R | GAAGAGAACGAAAGGAGAACAGA |  |
| nad5-F | CGCTCGAACATTGTCTGATT |  |
| nad5-R | GTCCTGGCAAGCTCCTACAG |  |
| nad6-F | TGGAAAAACCAAACCCACAT |  |
| nad6-R | CAAGTTCCCTTGGCGTAGTC |  |
| nad7-F | GTTTTGGCTCGCAATAAAGC |  |
| nad7-R | CAGGTGGGACAAGCTCTAGG |  |
| nad9-F | AGCAAGAAGCGGAACAAAAA |  |
| nad9-R | TATTGATTTGTCCCCTCCCC |  |
| cob-F | ATCAAGGCAAGGGGGTAAAT |  |
| cob-R | GGTGTGATCAGTCTCATCCG |  |
| cox1-F | GGCCCCTCTCTGATAAGGTT |  |
| cox1-R | GTTAAGGCAAAGCCCAAACA |  |
| cox2-F | GTCCTACTTCTGGTGCTGCC |  |
| cox2-R | GAGAATTGCATTTCCGCTTC |  |
| cox3-F | TCAATCCACTTATTCGTTCCC |  |
| cox3-R | GTTTACATACAACCGGGGCA |  |
| atp1-F | CGTTGCTGGTGAAGAAGCAT |  |
| atp1-R | AAAAGCGGATTTATCCATCG |  |
| atp4-F | AGCCACGTGCTCTAATCCTC |  |
| atp4-R | TCCCTTTCTCTTGGAGCAGA |  |
| atp6-F | CCAAGTCTCTTTTGGGAGCA |  |
| atp6-R | GGCTCCTCGTTTTTATGCAA |  |
| atp8-F | GGCAAGGATCCTCAGTCCTA |  |
| atp8-R | GAGGGTTGGTTTGATTGGAA |  |
| atp9-F | AGGGGCCTCGTCATCTCTAT |  |
| atp9-R | TAGTTGCGAAGGAAAAGCGT |  |
| ccmB-F | AGCCGTCGAAGTGAATGAAT |  |
| ccmB-R | AACGGCTTTTCCATGACTTG |  |
| ccmC-F | ACTTGCAAGGCAAGGAAAAA |  |
| ccmC-R | CCATGGATGCTTTAGCGAGT |  |
| ccmFc-F | GAGAAGCTCAAATCGAACGG |  |
| ccmFc-R | CGCAGCCACTATTTTGACTC |  |
| ccmFn-F | TGAAGATTGTAAGGCGTTTCC |  |
| ccmFn-R | GGATCATCCTGTGGTTACCG |  |
| rps1-F | AAGGTGGGCTTCGGATTATT |  |
| rps1-R | TCTTCAGTTTTACGCTTACGCT |  |
| rps2A-F | CAGGAAAGATATTTGCCCCA |  |
| rps2A-R | CCTGTATCTCCGGAAACGAA |  |
| rps2B-F | TCCATGGACCCACGTAAAAT |  |
| rps2B-R | GGCCCCTCTCTGATAAGGAA |  |
| rps3-F | GCAGAAAGGGGCAAAAGTAA |  |
| rps3-R | TCGCGACCCCTACTACATCT |  |
| rps4-F | AGAGTTGGGTTCGATTCCCT |  |
| rps4-R | AGCGACTAGGCCGATCTTTT |  |
| rps7-F | TTCGTTGGAAAAACCTACGC |  |
| rps7-R | ATGAGGAAGGCCGATTTTCT |  |
| rps7-ct-F | TTGAACCTCTTTCACGCTCA |  |
| rps7-ct-R | TTCCGATCGAGATGTATGGA |  |
| rps12-F | CTAGCTGCTTCCATATCGCC |  |
| rps12-R | CGGATCGGGAGTAACCACTA |  |
| rps12-ct-F | TGTACGGTTCTGTAGAGGGACA |  |
| rps12-ct-R | TCCGTTTTCTTTTTATAAGGGC |  |
| rps13-F | TCATGATGATTAAGGGAAGAGTGA |  |
| rps13-R | TTGAATTGAACAGTGTGATTGAT |  |
| rpl16-F | GGTTTTTCCCCACTAACCAA |  |
| rpl16-R | GGGTGCGGAAATAGCTAGAA |  |
| mat-r-F | AACGCCTGTTCGCTAAAATC |  |
| mat-r-R | AGGCTTTGCTCCCCTTTTT |  |
| mttB-F | TTGGTTTAGAATTGCTCGGG |  |
| mttB-R | AGGGGGAACCCTACCGAC |  |
| AD-Zmd049043F | CGGAATTCATGGCCCTCGCACTGCGCC | Yeast two-hybrid assay |
| AD-Zmd049043R | CGGGATCCTCATACCTGCCAGTTTCCTTG |  |
| AD-Zmd045528F | CGGAATTCATGGCCGCCGCCGCC |  |
| AD-Zmd045528R | CGGGATCCTCATCGCTGGTTCTCCCTC |  |
| AD-Zmd026307F | CGGAATTCATGGCAGCCGCATCAGCG |  |
| AD-Zmd026307R | CGGGATCCTCAGTGCTGCTCCTGATTC |  |
| AD-Zmd038760F | CGCATATGATGTATGCTTTTAGTACGAC |  |
| AD-Zmd038760R | CGGAATTCCTAGTCGCAGCTCCCAGTG |  |
| AD-Zmd026243F | CGGAATTCATGGCCACCGTAGCGCGC |  |
| AD-Zmd026243R | CGGGATCCTCATCTCTGGTAAGATTGG |  |
| AD-Zmd048291F | CGGAATTCATGGCGTCGGCGTCGCGTG |  |
| AD-Zmd048291R | CGGGATCCTCACTGGAAGTTGGAGTTGCC |  |
| AD-Zmd024674F | CGGAATTCATGGCCGCCTCCCTCCCGAC |  |
| AD-Zmd024674R | CGGGATCCTCACGAAGACGCGGACTCG |  |
| DEK55-BD-F | CGGAATTCGACCGCTACCTTGCTAACGCG |  |
| DEK55-BD-R | CGGGATCCTCAGATACCAAAATCACAAGC |  |
